# Social disadvantage accelerates aging

**DOI:** 10.1038/s41591-025-03563-4

**Published:** 2025-03-14

**Authors:** Mika Kivimäki, Jaana Pentti, Philipp Frank, Fangyu Liu, Acer Blake, Solja T. Nyberg, Jussi Vahtera, Archana Singh-Manoux, Tony Wyss-Coray, Keenan A. Walker, Linda Partridge, Joni V. Lindbohm

**Affiliations:** 1 Brain Sciences, University College London, London, UK; 2 Clinicum, University of Helsinki, Helsinki, Finland; 3 Department of Public Health and Centre for Population Health Research, University of Turku, Turku University Hospital, Turku, Finland; 4 Laboratory of Behavioral Neuroscience, National Institute on Aging, National Institutes of Health, Baltimore, MD USA; 5 Institute of Healthy Ageing, GEE, University College London, London, UK; 6 MPLS (Mathematical, Physical and Life Sciences) Division, Oxford University, Oxford, UK; 7 Inserm U1153, Epidemiology of Ageing and Neurodegenerative Diseases, Université Paris Cité, Paris, France; 8 Department of Neurology and Neurological Sciences, Stanford University School of Medicine, Stanford, CA USA; 9 Wu Tsai Neurosciences Institute, Stanford University, Stanford, CA USA; 10 The Phil and Penny Knight Initiative for Brain Resilience, Stanford University, Stanford, CA USA; 11 Broad Institute of MIT and Harvard University, Cambridge, MA USA

**Keywords:** Risk factors, Inflammation

## Abstract

Social disadvantage, like advanced age, is a risk factor for a broad range of health conditions; however, whether it influences the aging process remains unclear. Here, using a multicohort approach, we investigated the associations of social disadvantage with age-related plasma proteins and age-related diseases. We found proteomic signatures of accelerated immune aging and 14 specific age-related proteins linked to social disadvantage during both early and later life. Individuals experiencing social disadvantage had an increased risk of 66 age-related diseases, with up to 39% of these associations mediated by the 14 age-related proteins (for example, DNAJB9, F2, HSPA1A, BGN). The main enriched pathway involved the upregulation of the pro-inflammatory regulator NF-κB24 and its downstream factor interleukin-8. Our findings support the hypothesis that social disadvantage throughout the life course may accelerate aging, a biological mechanism that could explain why social stratification plays such a fundamental role in determining human health.

## Abstract (web summary)

Individuals with social disadvantage, during both early and later life, have an increased risk of 66 age-related diseases mediated by 14 age-related proteins.

## Main

Most human health conditions are socially patterned^1–3^. Social disadvantage is a known risk factor for mental and behavioral disorders, poor glucose and insulin metabolism, and impaired immune function, as well as diseases of the liver, kidney, and vascular and respiratory systems, site-specific cancers and dementia^3^. Such a breadth of health effects is rarely observed for a single risk factor, except age. As with social disadvantage, the progressive cellular damage associated with aging increases susceptibility to most diseases^4–6^.

Parallels between the effects of social disadvantage and biological aging suggest that social disadvantage may accelerate the aging process^7–9^. Aging is associated with declining metabolic function, including increased insulin resistance, impaired hepatic gluconeogenesis, dysregulated adipose lipogenesis and atherosclerosis^10,11^. Many components of the immune system also deteriorate with age. These changes include an increase in levels of circulating pro-inflammatory mediators and a downregulation of immune responsiveness^12,13^. Notably, the same changes are characteristic of the effects of social disadvantage^12,14–17^. In addition, there is evidence linking social disadvantage to biological aging, as measured by epigenetic aging clocks^7–9^.

If social disadvantage accelerates the aging process, it should be linked to one or more of the nine original hallmarks of cellular aging^4,5,18^. These include four primary contributors to age-related cellular damage—genomic instability (GI), telomere attrition (TA), epigenetic alterations (EA) and loss of proteostasis (LOP, the breakdown of the cellular mechanisms that regulate the proper synthesis, folding, trafficking and degradation of proteins). In addition, they comprise five compensatory or integrative hallmarks—deregulated nutrient sensing (DNS), mitochondrial dysfunction (MD), cellular senescence (CS), altered intercellular communication (AIC) and stem cell exhaustion (SCE)^5^. Although not diseases themselves, these hallmarks contribute to age-related conditions, such as SCE increasing cardiovascular risk, CS heightening susceptibility to infections and GI being linked to cancer^4,5,18^.

**Fig. 1:**
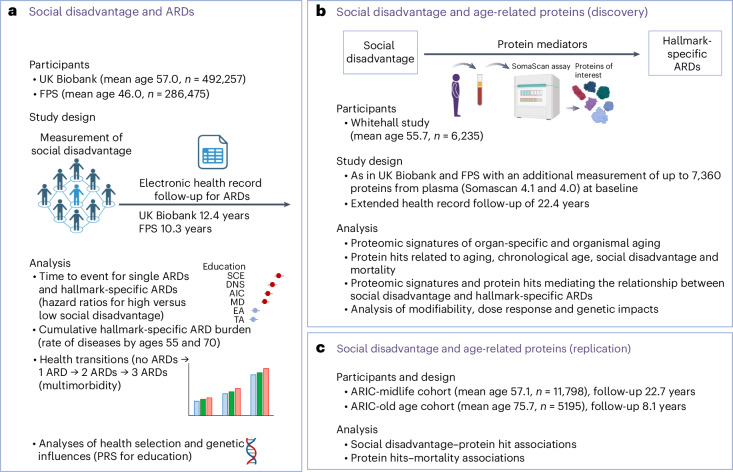
Overview of the study design. **a**, Summary of study characteristics and analyses from the UK Biobank and the FPS. Indicators of social disadvantage were education and adulthood SES, measured by residential neighborhood deprivation and occupational position. In individuals with social disadvantage, a higher risk of ARDs was observed across all nine hallmarks of aging. This was evident for the onset of the first ARD, cumulative ARD burden and ARD multimorbidity. By contrast, there was limited evidence for the reverse direction of the relationship, in which ARDs lead to social disadvantage, or for genetic factors explaining the observed associations. **b**, The characteristics and analyses of the Whitehall study, including proteomic data, are summarized. With the exception of immune function and kidney aging, proteomic signatures of organ-specific aging were not strongly associated with social disadvantage. By contrast, of the 1,040 hallmark-related proteins associated with chronological age at proteome-wide significance, 14 were consistently linked to indicators of social disadvantage and hallmark-specific ARDs, partially mediating this association. Additional analyses supported the modifiability of these proteins in relation to changes in social disadvantage and a dose–response association indicating accumulated risk. **c**, Characteristics and analyses of the two cohorts of the ARIC study, including data on 11 of the 14 proteins, are summarized. The associations between social disadvantage and protein levels replicated the findings from the Whitehall study. Similarly, the longitudinal associations between these proteins and all-cause mortality, with both 23-year and shorter 8-year follow-ups, were consistent with those observed in Whitehall. Figure created with BioRender.com.

To better understand the links between social disadvantage and biological aging, we examined whether social disadvantage is associated with 83 age-related diseases (ARDs) and over 3,900 age-related circulating proteins, including those linked to accelerated organ-specific and organismal aging^19,20^ (Fig. 1). Our findings support the concept that social disadvantage may accelerate age-related changes, aligning with the social causation hypothesis. While reverse causality—in which ARDs contribute to social disadvantage—and genetic predisposition influencing both the likelihood of social disadvantage and the development of ARDs were also observed, their roles appear more limited.

## Results

This study draws on four prospective observational studies, comprising five cohorts analyzed sequentially. Two large-scale studies, the UK Biobank and the Finnish Public Sector Study (FPS), were used to test the social causation hypothesis in relation to ARDs associated with hallmarks of aging (Fig. 1a). We tested the alternative health-related selection hypothesis using the FPS, with its repeated measures of social disadvantage, to examine whether ARDs increase the risk of social disadvantage. In addition, genetic data from the UK Biobank enabled an investigation into the genetic contributions to these associations^21^. To identify age-related protein mediators of the associations between social disadvantage and hallmark-specific ARDs, we used proteomic data from the UK Whitehall study (Fig. 1b) and replicated the main findings in the US Atherosclerosis Risk in Communities (ARIC) study, including both midlife and older-age cohorts (Fig. 1c).

**Extended Data Fig. 1:**
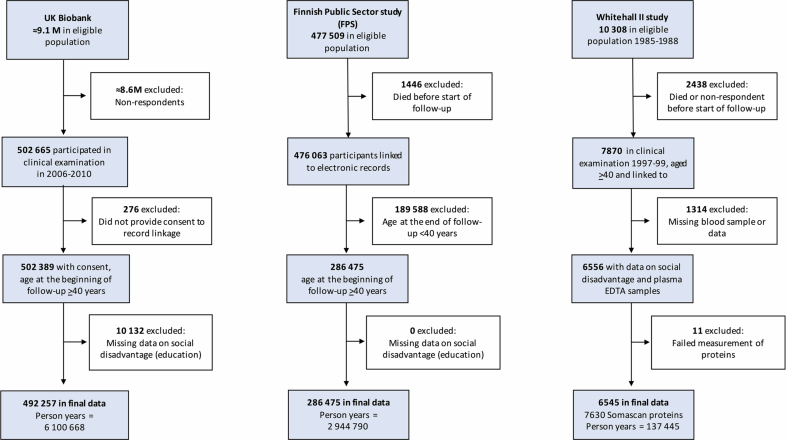
**Fig. 1. Sample selection for the cohorts in the primary analysis**. Source data

**Extended Data Fig. 2:**
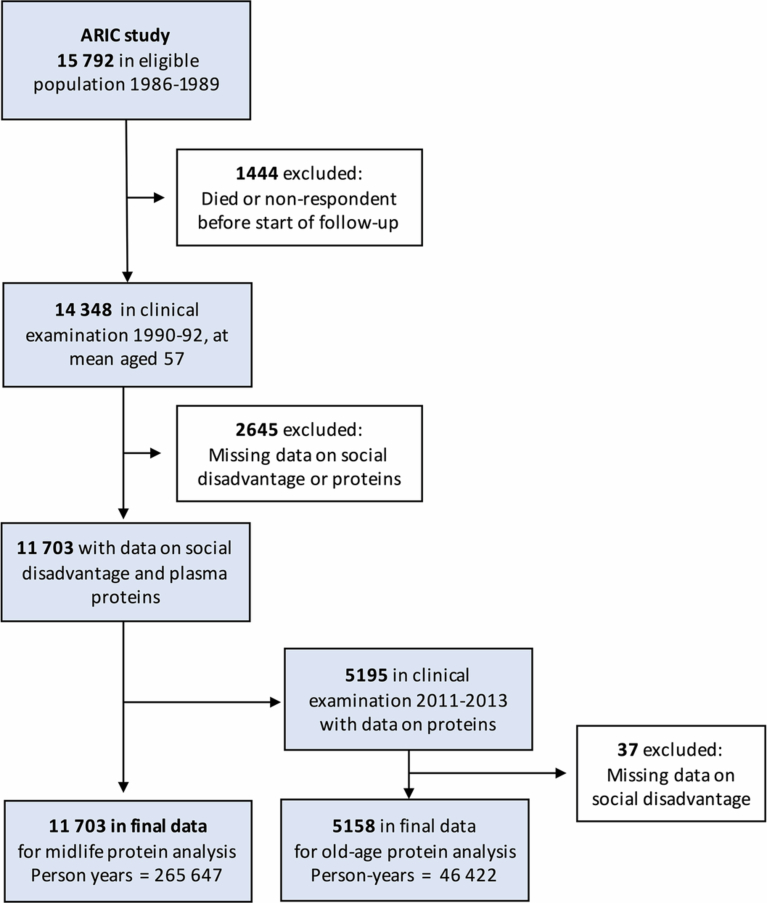
**Fig. 2 Sample selection for the cohorts in the replication study**. Source data

In these four studies, social disadvantage was measured in both early and later life (Table 1, Extended Data Figs. 1 and 2, and Supplementary Table 1). Education, typically spanning ages 5–7 to ages 15–25, was assessed in all cohorts as a marker of social standing in early life and across the life course. In the Whitehall study, father’s social class—representing influences from birth until independent living—served as an additional early-life indicator. In all cohorts, neighborhood deprivation was used as a measure of adult socioeconomic status (SES). Other indicators of adult SES included occupational position in the FPS and Whitehall cohorts, and family income in the ARIC cohorts.

**Table 1:** Baseline characteristics of participants by cohort

Characteristics	UK Biobank		FPS		Whitehall		ARIC midlife		ARIC old age	
	*n*	%	*n*	%	*n*	%	*n*	%	*n*	%
Number of participants	492,257	100	286,475	100	6,544	100	11,798	100	5,195	100
Age (years), mean (s.d.)	57.0 (8.1)		46.0 (7.4)		55.7 (6.0)		57.1 (5.7)		75.7 (5.2)	
Sex										
Men	224,286	45.6	76,902	26.8	4,641	70.9	5,242	44.4	2,970	57.2
Women	267,971	54.4	209,573	73.2	1,903	29.1	6,556	55.6	2,225	42.8
Ethnicity										
White	464,816	94.4			5,982	91.4	8,987	76.2	4,200	80.8
Non-White	25,768	5.2			562	8.6	2,733	23.2	966	18.6
Missing	1,673	0.3			0	0.0	78	0.7	29	0.6
PRS for education										
Mean (s.d.)	0.0 (1.0)	97.7			0.0 (1.0)	48.9				
Missing	11,550	2.3			3,187	51.1				
**Early-life social indicator**										
Education										
Primary	85,258	17.3	34,693	12.1	2,336	35.7	2,564	21.7	700	13.5
Secondary	245,883	50.0	96,917	33.8	1,736	26.5	4,948	41.9	2,192	42.2
Tertiary	161,116	32.7	154,865	54.1	2,339	35.7	4,269	36.2	2,295	44.2
Missing	0	0.0	0	0.0	133	2.0	17	0.1	8	0.2
**Adult SES indicators**										
Neighborhood deprivation										
Low	124,225	25.2	89,606	31.3	1,575	24.1	4,139	35.1	1,735	33.4
Intermediate	245,783	49.9	148,197	51.7	3,167	48.4	3,538	30.0	1,735	33.4
High	121,637	24.7	37,970	13.3	1,513	23.1	3,787	32.1	1,712	33
Missing	612	0.1	10,702	3.7	289	4.4	334	2.8	13	0.3
Occupational status or income										
Low			79,431	27.7	863	13.2	3,987	33.8	1,870	36.0
Intermediate			74,090	25.9	2,801	42.8	4,283	36.3	1,599	30.8
High			120,194	42.0	2,806	42.9	2,882	24.4	1,238	23.8
Missing			12,760	4.5	74	1.1	646	5.5	488	9.4

For additional indicators of social disadvantage, see Supplementary Table 1.

### Social disadvantage and risk of ARDs

As an initial step, we selected ARDs associated with hallmarks of aging. These included a total of 83 diseases linked to one or more hallmarks of aging, based on the taxonomy put forward in ref. ^4^ (Supplementary Table 2). Support for this taxonomy comes from multiple sources. Analyses of electronic health records from general practice and hospitalizations identified more than 200 diseases with incidence rates increasing with chronological age^6,22^. Researchers linked a subset of these ARDs to specific hallmarks of aging using several approaches: mining 1.85 million PubMed abstracts on human aging, identifying shared genes in the genome-wide association study catalog, conducting gene set enrichment analysis and analyzing disease co-occurrence networks within each hallmark^4^.

**Extended Data Fig. 3:**
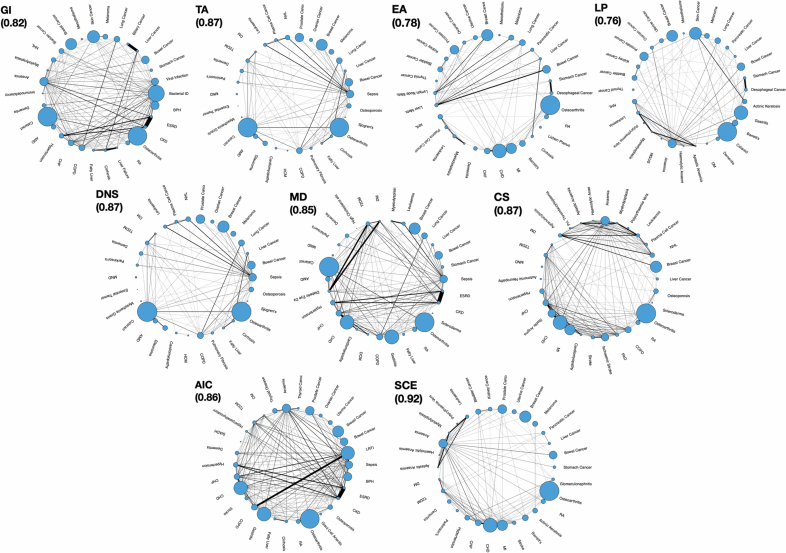
Fig. 3. Barrat Global Network Transitivity Analysis of disease associations in hallmark-specific ARDs (source: Kivimaki et al. Lancet Healthy Longevity 2024^23^). Data are from the UK Biobank study (total N = 492,257). Number in parenthesis is clustering coefficient. Circle size is proportional to the occurrence of the disease. AIC, altered intercellular communication; ARD, age-related disease; CS, cellular senescence; DNS, deregulated nutrient sensing; EA, epigenetic alterations; GI, genomic instability; LOP, loss of proteostasis; MD, mitochondrial dysfunction; SCE, stem cell exhaustion; TA, telomere attrition.

**Extended Data Fig. 4:**
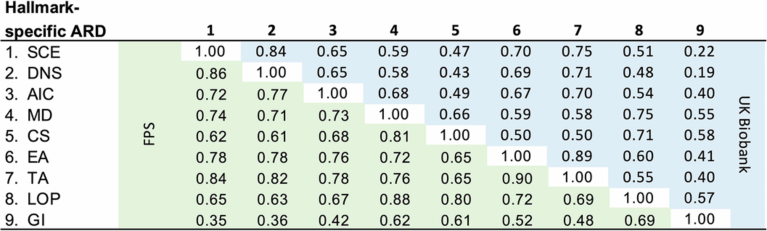
Fig. 4. Correlation matrix for hallmark-specific ARDs in the UK Biobank and the Finnish Public Sector studies. Numbers are Phi coefficients of bivariate correlations between hallmark-specific ARDs in the UK Biobank (total N = 492,257) and Finnish Public Sector (total N = 286,475) studies. AIC, altered intercellular communication; ARD, age-related disease; CS, cellular senescence; DNS, deregulated nutrient sensing; EA, epigenetic ageing; FPS, Finnish Public Sector study; GI, genomic instability; LOP, loss of proteostasis; MD, mitochondrial dysfunction; SCE, stem cell exhaustion; TA, telomere attrition. Source data

We confirmed the co-occurrence of ARDs within each hallmark in 492,257 participants from the UK Biobank study^23^. The presence of one ARD increased the risk of developing another ARD related to the same hallmark, with clustering coefficients ranging from 0.76 for LOP-specific ARDs to 0.92 for SCE-specific ARDs. These findings corroborated the hallmark-specific clustering of ARDs (Extended Data Figs. 3 and 4)^23^.

In time-to-event analyses of UK Biobank and FPS participants without these ARDs at baseline (*n* ranging from 477,325 to 492,294 in the UK Biobank and from 278,272 to 286,471 in the FPS, depending on the social disadvantage indicator and ARD), social disadvantage—indicated by education and adult SES (neighborhood deprivation)—was associated with a higher risk of developing ARDs. In the UK Biobank, the age-, sex- and ethnicity-adjusted hazard ratio for developing any ARD was 1.31 (95% confidence interval (CI) 1.29–1.33) for individuals with low compared with high education. For individuals with high versus low adult SES, the hazard ratio was 1.21 (95% CI 1.20–1.23). In the FPS, the corresponding hazard ratios were 1.28 (95% CI 1.25–1.31) and 1.23 (95% CI 1.20–1.27), respectively.

**Fig. 2:**
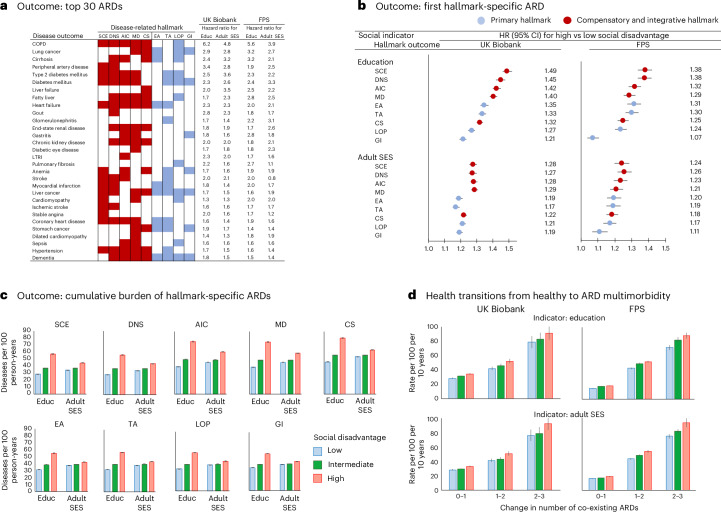
Social disadvantage and risk of diseases associated with hallmarks of aging. These analyses examine the social causation hypothesis using two-sided tests without adjustments for multiple comparisons (for full results, see Supplementary Tables 3–12). The whiskers represent 95% CIs. **a**, The numbers indicate hazard ratios from Cox proportional hazards models comparing high versus low social disadvantage at baseline (education and adulthood SES), adjusted for age, sex and ethnicity, for a single ARD risk at follow-up in the UK Biobank and FPS (sample size 481,197–492,257 in the UK Biobank and 275,157–285,830 in the FPS, depending on the social disadvantage indicator and ARD). All HRs are statistically significant (*P* < 0.05). Only the 30 strongest associations are shown. **b**, The forest plot shows hazard ratios from Cox models for developing hallmark-specific ARDs, comparing participants with high versus low social disadvantage in a population free of these diseases at baseline. All HRs are statistically significant (*P* < 0.05). In the UK Biobank, sample sizes ranged from 430,373 to 460,980, with the corresponding FPS sample sizes between 257,073 and 281,348. **c**, The bars show the number and 95% CIs of hallmark-specific ARDs per 100 person-years by age 70 in the UK Biobank, stratified by social disadvantage level, estimated using Poisson regression (range of person-years 34,134,089–34,174,830). The results for FPS are available in Supplementary Table 7 (range of person-years 15,508,103–16,112,263). **d**, The bars represent ARD multimorbidity progression rates, from an ARD-free state to three co-occurring ARDs, stratified by social disadvantage levels, based on Poisson regression analysis in a population free of these diseases at baseline (range of person-years 4,959,308–4,965,499 in UK Biobank and 2,614,663–2,712,578 in FPS). HR, hazard ratio; Educ, education. Source data

**Extended Data Fig. 5:**
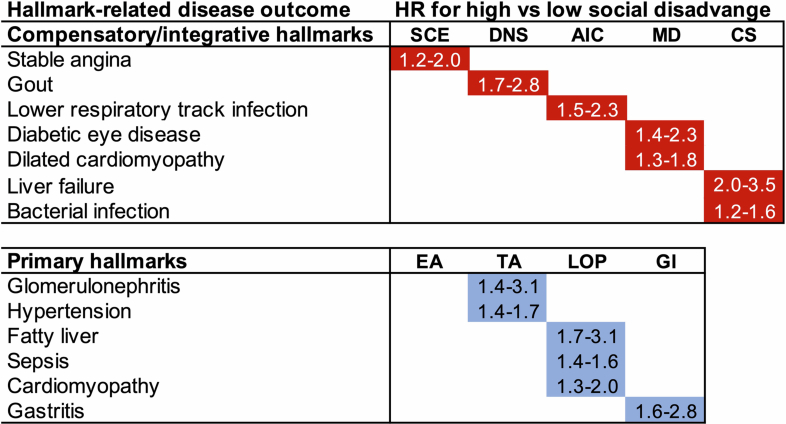
Fig. 5. Cox proportional hazards regression analysis of social disadvantage and risk of single hallmark-specific ARDs. Numbers represent the range of statistically significant age-, sex-, and ethnicity-adjusted hazard ratios for high versus low social disadvantage (education, neighbourhood deprivation, occupational position) in relation to specific ARDs in the UK Biobank (N = 492,257) and the Finnish Public Sector study (N = 286,475). These ranges reflect associations where ARDs are linked exclusively to a single compensatory/integrative hallmark or a single primary hallmark of aging. Full results for all social disadvantage-ARD associations are available in Supplementary Table 3. AIC, altered intercellular communication; ARD, age-related disease; CS, cellular senescence; DNS, deregulated nutrient sensing; EA, epigenetic ageing; GI, genomic instability; HR, hazard ratio; LOP, loss of proteostasis; MD, mitochondrial dysfunction; SCE, stem cell exhaustion; TA, telomere attrition. Source data

Overall, social disadvantage was associated with an increased risk of 66 specific ARDs (Supplementary Table 3). The 30 ARDs most strongly associated with social disadvantage were multi-hallmark diseases, as classified by the taxonomy in ref. ^4^ (Fig. 2a). These included liver, metabolic, circulatory, respiratory, infectious and neurodegenerative diseases. By contrast, social disadvantage was less strongly associated with diseases linked to a single primary or compensatory and integrative hallmark of aging (Extended Data Fig. 5).

**Extended Data Fig. 6:**
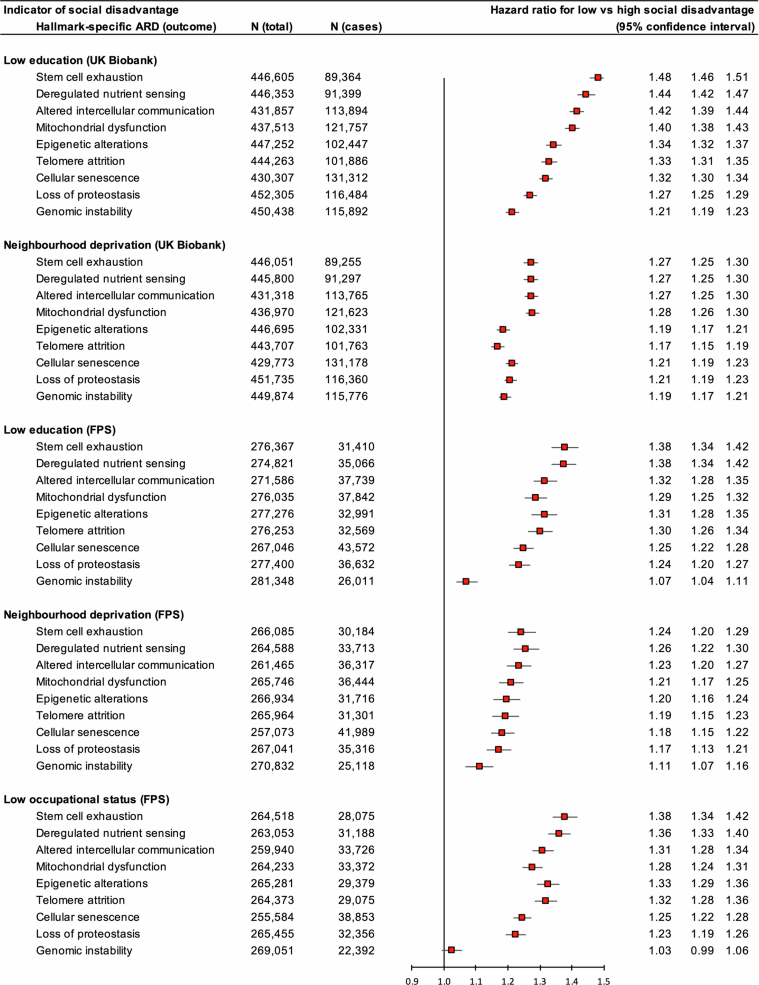
Fig 6. Time-to-event analysis for social disadvantage and developing the first hallmark-specific ARDs in participants with no such diseases at the time social disadvantage was measured. Data are from the UK Biobank and the Finnish Public Sector study. The plots show the age-, sex- and ethnicity-adjusted hazard ratios for hallmark-specific ARDs in participants with high versus low social disadvantage, based on Cox proportional hazards regression analysis. Whiskers show 95% confidence interval for these estimates. AIC, altered intercellular communication; ARD, age-related disease; CS, cellular senescence; DNS, deregulated nutrient sensing; EA, epigenetic ageing; FPS, Finnish Public Sector study; GI, genomic instability; HR, hazard ratio; LOP, loss of proteostasis; MD, mitochondrial dysfunction; SCE, stem cell exhaustion; TA, telomere attrition. Source data

In separate time-to-event analyses of the first-onset ARD within each of the nine hallmarks, individuals with social disadvantage had a higher risk of ARDs across all hallmarks compared with those without social disadvantage (Extended Data Fig. 6 and Supplementary Table 4). In both the UK Biobank and FPS, these associations were generally stronger for ARDs linked to compensatory and integrative hallmarks than for those associated with primary hallmarks (Fig. 2b). This was particularly evident for SCE-, DNS-, AIC- and MD-specific ARDs, while the weakest association was observed for GI-specific ARDs. These findings were robust, as similar patterns were observed in analyses using an alternative indicator of adult SES—occupational position—available for FPS participants and in both men and women, although the effects were stronger in men (Supplementary Tables 4 and 5).

Given the higher mortality rate among individuals with social disadvantage, we examined whether competing risks, such as deaths, might bias the associations between social disadvantage and the risk of hallmark-specific ARDs. However, no such evidence was found, as these associations persisted in competing risk analyses with hallmark-specific ARDs and death as outcomes (Supplementary Table 6).

To determine whether social disadvantage was associated with the total hallmark-specific ARD burden, including both first and subsequent ARDs, we calculated the average number of hallmark-specific ARDs per 100 person-years by age 70 in participants from the UK Biobank. The age-, sex- and ethnicity-adjusted rate was up to two times higher in individuals with greater social disadvantage than in those with less social disadvantage (Fig. 2c and Supplementary Tables 7 and 8). A similar pattern was observed when estimated up to age 55 in the younger FPS cohort compared with the UK Biobank population (Supplementary Tables 7 and 8). These findings were consistent across both indicators of social disadvantage—education and adult SES—in both cohorts, as well as for occupational positions in FPS participants.

To examine each health transition separately, we conducted a stepwise analysis focusing on the development of multiple ARDs during the follow-up, starting from a baseline healthy state with no ARDs. Transition rates from 0 to 1, 1 to 2 and 2 to 3 ARDs increased by 1.5 to more than 2 times at each step, with the highest rates consistently observed in the socially disadvantaged group at every transition (Fig. 2d and Supplementary Tables 9 and 10).

Overall, these results support a social causation model, suggesting that social conditions in both early and later life influence the risk of ARDs and contribute to health disparities across groups defined by educational background, residential neighborhoods and occupational status. In a model including both education and adult SES, each indicator showed an independent association with ARDs with the effect of education being stronger than that of neighborhood deprivation (Supplementary Table 11).

### Health-related selection into social disadvantage

We then examined the reverse causality direction, wherein ARDs are hypothesized to negatively impact subsequent levels of adult SES, a concept known as the health-related selection model. Among FPS participants whose adult SES changed between baseline and follow-up, those with an ARD at baseline were more likely to experience a downward shift in adult SES compared with participants without an ARD at baseline (Supplementary Table 12). However, the effect estimates—indicating a 1.1 to 1.2 times higher risk of declining SES over the next 5 years (*n* = 58,000) or 10 years (*n* = 39,608) for individuals with preexisting ARD—were weaker than those supporting the social causation model. Thus, while both social causation and health-related selection processes are probably at play, social causation appears to have a stronger influence.

### Social disadvantage and age-related circulating proteins

ARDs represent outcomes of aging rather than the aging process itself. To better understand the aging process in relation to social disadvantage, beyond its outcomes, we examined the association between social disadvantage and blood-based proteomic signatures of aging. Our rationale for these analyses is threefold. First, aging is associated with measurable changes in the plasma proteome^19,20^. Circulating proteins regulate the aging process, while aging also modifies blood protein composition. Some proteins additionally serve as markers of aging, entering the plasma through leakage from damaged or dying cells. Second, ARDs and age-related proteins are empirically distinct constructs: the former are based on hospitalization diagnoses, and the latter on biomarker measurements, together providing a complementary assessment of aging from two independent information sources. Third, proteins reflect multiple age-related processes that precede ARDs. Analyzing them helps identify specific aging processes influenced by social disadvantage and the extent to which these processes mediate the associations of social disadvantage with individual aging outcomes.

The Whitehall study is one of the few long-term prospective cohort studies that provide extensive data on indicators of social disadvantage, link participants to electronic health records for assessing hallmark-specific ARDs and store plasma samples for biomarker measurements collected between 1997 and 1999, coinciding with the assessment of social disadvantage. In addition, consistent with findings from the UK Biobank and FPS, education and adult SES (including neighborhood deprivation and occupational position) were associated with hallmark-specific ARDs in the 22-year follow-up of the Whitehall study (Supplementary Table 13).

To analyze differences in age-related plasma proteins among 6,235 Whitehall participants with high and low levels of social disadvantage (mean age 56, 29% women), we assessed expression levels of up to 7,360 circulating proteins using the SomaLogic proteomic platform assays version 4.0 (*n* participants = 2,213) and version 4.1 (*n* = 4,022). Using the Organage package for Python^24^, we estimated proteomic signatures for the biological age of each participant’s organs relative to those of same-aged peers, that is, organ age gaps, as well as a non-organ-specific (organismal) age gap^24^ (Supplementary Table 14).

**Fig. 3:**
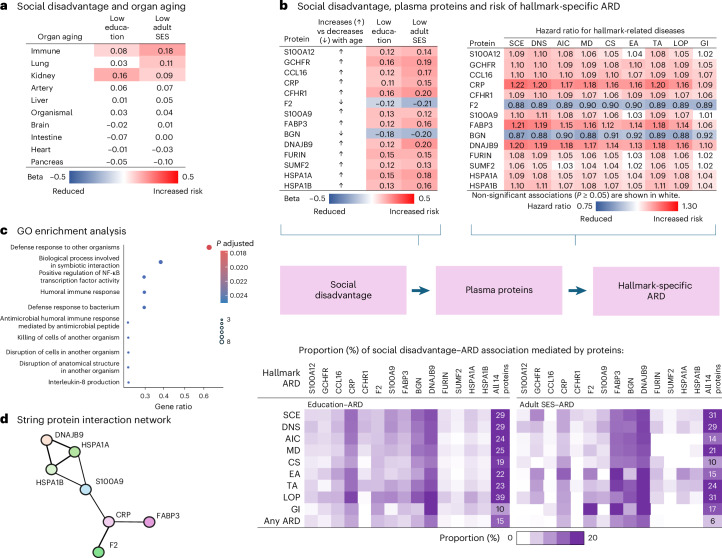
Plasma proteins associated with social disadvantage and risk of hallmark-specific ARDs and mortality. **a**,**b**, Findings from the Whitehall study; full results are in Supplementary Tables 15–24. **a**, The heat map shows results from multinomial logistic regression analyses examining the associations between social disadvantage and protein signatures of age gaps defined as the biological age of an individual’s organs or body relative to that of same-aged peers. The numbers represent beta coefficients for low versus high education and neighborhood deprivation, adjusted for age, sex and ethnicity. **b**, Of the 1,040 proteins with concentrations significantly associated with chronological age at the proteome-wide level (two-sided *P* < 1.67 × 10^−6^), 14 proteins are highlighted for their consistent associations with age (linear regression analysis), social disadvantage indicators (cumulative logistic regression analysis) and mortality (Cox regression). The numbers in the upper heat maps show beta coefficients and hazard ratios per s.d. increase in protein concentration, from linear regression for social disadvantage and Cox regression for ARDs, adjusted for age, sex and ethnicity. The lower heat map shows the proportion of the association between social disadvantage and hallmark-specific ARDs mediated by the 14 proteins, calculated using the inverse odds ratio-weighted method. **c**, GO enrichment analyses of the 14 proteins are shown. Rows show the GO terms, the dot sizes show the number of enriched proteins, the colors indicate the FDR-adjusted *P* value and the *x* axis shows the proportion of enriched proteins relative to all proteins associated with the GO term. **d**, String protein interaction network analysis indicates that 7 of the 14 proteins formed a protein interaction network. Only associations above a confidence score of 0.4 (standard medium confidence) are shown. Source data

**Extended Data Fig. 7:**
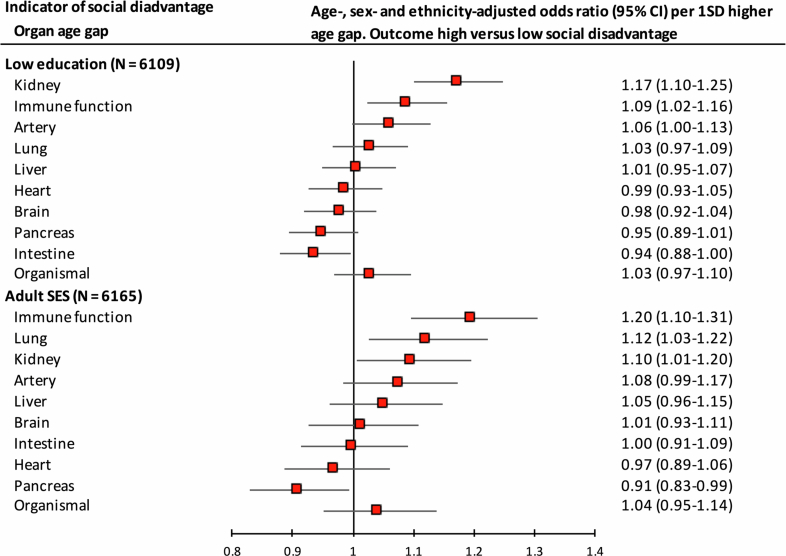
Fig. 7. Cross-sectional associations between indicators of social disadvantage and plasma proteomic signatures of organ-specific and organismal (non-organ-specific) age gaps. Data are from the Whitehall study. The plots display the odds ratios for low vs high social disadvantage from multinomial logistic regression analyses for organ-specific and organismal age gaps, defined as the biological age of an individual’s organs or body relative to that of same-aged peers. Whiskers show 95% confidence interval for these estimates. Source data

After adjustment for age, sex and ethnicity, individuals with social disadvantage had higher age gaps in immune function, kidneys and lungs (Fig. 3a, Extended Data Fig. 7 and Supplementary Table 15). However, organ-specific age gaps mediated only a modest proportion of the associations between education and hallmark-specific ARDs (0% to 6.2%), and between adult SES and hallmark-specific ARDs (5.4% to 13.9%) (Supplementary Table 16).

To identify additional and potentially stronger mediators linking social disadvantage to hallmark-specific ARDs, we performed a higher-resolution protein-level discovery analysis. This analysis focused on 1,040 proteins of the 7,360 SomaLogic platform, which were previously linked to one or more hallmarks of aging in the Human Proteomic Atlas (www.proteinatlas.org)^4^ (Supplementary Table 17) and for which the expression levels in plasma were associated with chronological age at proteome-wide significance in the Whitehall study (*P* < 1.67 × 10^−6^) (Supplementary Table 18). These proteins are derived from a source of information independent of the health records used to assess hallmark-specific ARDs, ensuring that their use in mediation analyses is methodologically sound.

We found 14 hallmark-related proteins that were consistently associated with age, indicators of social disadvantage and mortality (Fig. 3b and Supplementary Tables 19 and 20). Furthermore, these proteins were associated with six to nine hallmark-specific ARDs, depending on the protein under investigation (Fig. 3b and Supplementary Table 21). Sex-stratified analyses showed a similar overall pattern of results in both men and women (Supplementary Table 22).

### Protein mediators of social disadvantage-related disease risk

To quantify the contributions of the 14 proteins to the associations between social disadvantage and hallmark-specific ARDs, we conducted a series of mediation analyses. The 14 proteins collectively mediated 9.6% to 39.2% of the associations between education and hallmark-specific ARDs, and 9.9% to 30.6% of the associations between adult SES and hallmark-specific ARDs, depending on the hallmark-specific ARD group of interest (Fig. 3b and Supplementary Tables 23 and 24). For ARDs associated with SCE, DNS, MD, TA and LOP, the proportion of mediation exceeded 20.0%. Separate analyses identified DNAJB9, CRP and BGN as the strongest mediators for education, and DNAJB9, FABP3 and BGN (followed by CRP and F2) for adult SES, in their associations with hallmark-specific ARDs.

**Extended Data Fig. 8:**
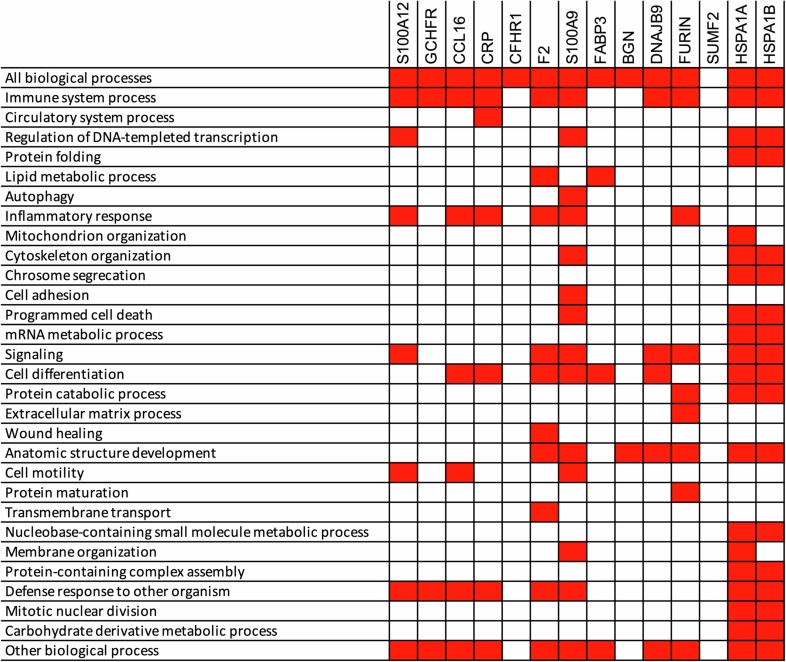
Fig. 8. Biological processes associated with the 14 mediator proteins linking social disadvantage and hallmark-specific ARDs, as identified from the Universal Protein Resource (UniProt, a comprehensive, freely accessible database of protein sequence and functional information). UniProt descriptions of biological processes for mediator proteins of the associations between social disadvantage and hallmark-specific diseases. Source data

To facilitate biological interpretation, we performed pathway enrichment analysis for the 14 proteins, revealing their enrichment in processes activated in response to both internal and external inflammatory stimuli. The main enriched inflammatory pathway was the upregulation of the pro-inflammation regulator NF-κB (ref. ^25^) and its downstream factor interleukin-8, which is associated with cell senescence^26^ (Fig. 3c and Extended Data Fig. 8). The related interaction network, formed by 7 of the 14 proteins, consisted of damage-associated molecular patterns (DAMPs) from the S100 protein family (S100A12 and S100A9), heat shock protein family (DNAJ9B, HSPA1A, HSPA1B) and C-reactive protein, which can recognize both DAMPs and pathogen-associated molecular patterns (PAMPs) (Fig. 3d).

### Protein concentrations reflect changes in social standing

We conducted additional analyses to assess whether the Whitehall data align with the social causation model regarding the associations between social disadvantage and the 14 proteins. First, if social disadvantage affects protein concentrations, the elimination of social disadvantage should relate to more favorable protein concentrations compared with persistent social disadvantage. Conversely, a shift from no social disadvantage to social disadvantage should be associated with less favorable protein concentrations (that is, concentrations related to a higher risk of ARDs) compared with a consistent absence of social disadvantage.

**Fig. 4:**
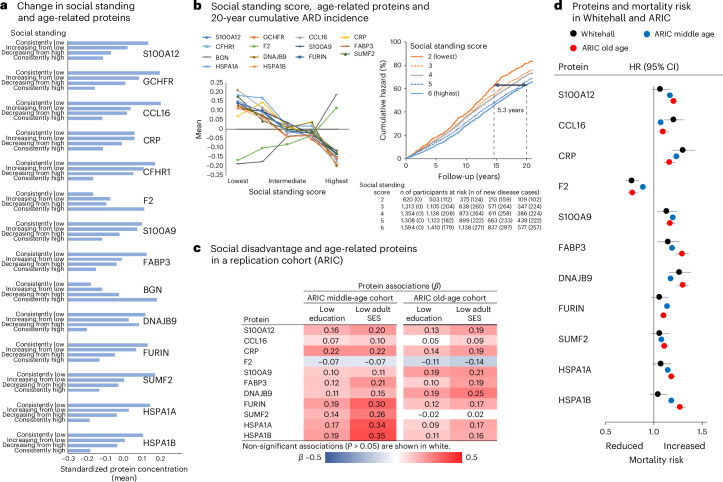
Supplementary and replication analyses for the associations between social disadvantage, protein concentrations, ARDs and mortality. These results from analyses examining the social causation hypothesis are based on two-sided tests, without adjustment for multiple testing (for full results, see Supplementary Tables 25–30). **a**, The *y* axis shows age-, sex- and ethnicity-adjusted means of standardized protein concentrations by combinations of early- and later-life social disadvantage categories, measured by education and adult SES in the Whitehall study. Supporting modifiability, a reduction in social disadvantage was associated with more favorable protein concentrations in later life, compared with persistent social disadvantage. Conversely, the onset of social disadvantage was linked to less favorable protein concentrations, compared with remaining free from disadvantage. **b**, The left panel shows adjusted means of standardized protein concentrations across a life-course social standing score (range: 2–6), illustrating dose–response associations: a higher social standing corresponds to more favorable protein concentrations, while lower scores indicate less favorable concentrations. The right panel shows cumulative hazard curves for ARDs during follow-up, stratified by baseline life-course social standing scores. Curve separation by social standing scores, derived from cumulative logistic regression, became apparent after baseline and widened over the 20-year follow-up. The incidence of ARD observed in individuals with the highest social standing score at follow-up year 20 was reached 5.3 years earlier in those with the lowest score. **c**, Age-, sex- and ethnicity-adjusted *β* coefficients from cumulative logistic regression analysis for the 11 proteins available in the ARIC study confirmed the associations with social disadvantage observed in the Whitehall study when analyzing midlife protein concentrations. With two exceptions, these associations were also evident in the analysis of protein levels measured in old age. **d**, The forest plot shows age-, sex- and ethnicity-adjusted hazard ratios per 1 s.d. higher protein concentration for mortality, based on Cox proportional hazards regression analyses. The whiskers represent 95% CIs. Source data

In the Whitehall study, this was supported in analyses combining early- and later-life indicators of social disadvantage (Fig. 4a and Supplementary Table 25). An improvement in social standing (that is, from low education to intermediate or high adult SES) was linked to more favorable protein concentrations compared with remaining in a low position (low education and low adult SES). A downward shift in social standing (from high education to intermediate or low adult SES) was associated with less favorable protein concentrations compared with remaining in a high position (high education and high adult SES).

### Evidence for dose–response relationships

To further investigate social causation, we examined whether a dose–response relationship existed between exposure to social disadvantage and the levels of age-related proteins. A graded association—in which greater social disadvantage corresponds to less favorable expression of age-related proteins—would support the social causation hypothesis. To quantify social disadvantage exposure for each Whitehall participant, we constructed a life course social standing score (ranging from 2 to 6) by summing the 3-level education and the 3-level adult SES variables (low = 1, intermediate = 2, high = 3). The subsequent risk accumulation analysis supported the social causation model, showing a dose–response relationship across all 14 proteins, with higher social standing scores linked to more favorable protein concentrations (Fig. 4b and Supplementary Table 26).

To evaluate whether a similar dose–response relationship exists for aging outcomes, we examined the association between the life course social standing score and the risk of developing ARDs during the follow-up period. Consistent with the social causation model, we found a dose–response association with ARDs, similar to that observed with protein concentrations. Separation in the hazard curves between groups with different social standing scores emerged shortly after baseline and widened throughout the entire follow-up period (Fig. 4b). Notably, the 20-year incidence of ARDs observed in individuals with the highest social standing score was reached more than 5 years earlier among those with the lowest score, highlighting age-related acceleration in ARD incidence in the latter group.

### Replication of protein associations in an independent cohort

To examine whether the findings on plasma proteins were replicable in an independent study population, we repeated the main analyses using data from the ARIC study. Like the Whitehall study, the ARIC study includes data on indicators of social disadvantage—such as education, neighborhood deprivation and family income—alongside protein measurements using the SomaLogic platform, with long-term follow-up for all-cause mortality. In ARIC, the SomaScan 4.0 assay measured 11 of the 14 proteins, with data collected during midlife (mean age 57.1) for 11,798 participants and in older age (mean age 75.7) for 5,195 participants (Supplementary Tables 27 and 28). These proteins were S100A12, CCL16, CRP, F2, S100A9, FABP3, DNAJB9, FURIN, SUMF2, HSPA1A and HSPA1B.

Results from education and adult SES in midlife (neighborhood deprivation) in ARIC replicated all associations with plasma proteins (Fig. 4c and Supplementary Table 29). At older ages, the findings were directionally consistent for these proteins, with the exception of SUMF2, which was not associated with social disadvantage in the ARIC cohort (Supplementary Table 30). In addition, the associations between proteins and mortality were directionally similar in the Whitehall study (follow-up of 22.4 years, s.d. 2.6) and the ARIC study, including the middle-age cohort with a mean follow-up of 22.7 years (s.d. 8.4) and the older cohort with a mean follow-up of 8.1 years (s.d. 2.5) (Fig. 4d).

### Test of genetic explanations

In addition to social causation and health-related selection (reversed causation), genetic predisposition may also drive the associations between social disadvantage, protein levels and hallmark-specific ARDs. To test this, we used an established polygenic risk score (PRS) for education^21^, available in the UK Biobank and Whitehall studies. Adjusting for this PRS, along with age, sex and ethnicity, had little impact on the associations of education and adult SES with hallmark-specific ARDs in these cohorts (Supplementary Tables 31 and 32). Similarly, the associations between education, adult SES and the 14 proteins in the Whitehall study remained robust following this adjustment (Supplementary Table 33). This was also the case for the associations between the proteins and hallmark-specific ARDs (Supplementary Table 34).

These findings indicate that genetic variation linked to social disadvantage has only a modest impact on the relationships between social disadvantage, age-related proteins and hallmark-specific ARDs.

## Discussion

This multicohort analysis of social disadvantage in relation to circulating proteins, ARDs and multimorbidity suggests that social disadvantage adversely affects a web of primary, compensatory and integrative hallmarks of aging, modifying multiple biological systems rather than specific tissues or organs. The over-twofold increased risk of type 2 diabetes, liver disease, heart failure, stroke, peripheral artery disease, gout, chronic obstructive pulmonary disease, lung cancer and mesothelioma in individuals with social disadvantage highlights the strong link between social disadvantage and hallmark-related morbidity. Furthermore, the 14 identified age-related circulating proteins mediated up to 39% of the social-disadvantage-related excess risk of hallmark-specific ARDs. These proteins were enriched in immune system responses to internal and external stimuli, with key downstream pathways via overactivation of NF-κB and IL-8. Changes in social disadvantage altered the levels of these proteins, suggesting that they may be modifiable.

Our study examines organ-specific proteomic aging in relation to social disadvantage, identifies over 1,000 plasma proteins potentially linked to the hallmarks of aging and investigates 83 hallmark-related diseases associated with social disadvantage and their protein mediators. The relationship between social disadvantage and hallmark-specific ARDs was robust, consistently observed in three independent cohort studies in the United Kingdom and Finland and replicated across indicators of both early- and later-life social disadvantage. Consistent findings from protein analyses in long-term follow-up studies in the United Kingdom and the United States further strengthen this evidence.

The validity of our study is supported by concordant findings from well-characterized, smaller-scale studies focused on a subset of aging hallmarks^25–31^. They suggest socioeconomic gradients in DNA methylation clocks^7–9^, associations between low education and TA^27–30^ and a link between low social rank and EA in mouse livers^31^. The overactivation of the NF-κB–IL-8 pathway identified in this study is associated with ARDs across multiple organ systems, potentially linking social disadvantage to hallmarks of aging^25,26,32^. Consistent with our findings, ‘inflamm-aging’^12^—a term denoting the accumulation of non-resolving low-grade inflammation—has been proposed as an additional meta-cellular hallmark of aging, interconnected with other aging hallmarks such as GI, EA, LOP, MD and CS^33^.

We found a modest contribution of accelerated organ-specific aging to the excess risk of ARDs in social disadvantaged individuals. However, this finding should be interpreted cautiously, as organ-specific aging was assessed using plasma protein signatures based on fewer than 120 proteins highly expressed in the specific organ^24^. Organ aging may involve processes outside the studied organs or processes not captured by the measured organ-specific gaps. Due to these limitations, we may have overlooked some socially patterned proteins contributing to accelerated organ aging.

The upstream drivers of the observed associations between social disadvantage, age-related proteins and ARDs remain unknown but probably include factors such as unhealthy behaviors^34,35^; life stress^11^; environmental exposures, such as pollutants and toxins^36^; and depression^37^. Research indicates that individuals experiencing social disadvantage are more likely to engage in smoking, heavy alcohol consumption and drug abuse; consume an energy-dense nutrient-poor diet; show physical inactivity; experience disturbed sleep; neglect safety behaviors (for example, self-examination, participation in screening and vaccination); and fail to adhere to medical procedures (for example, antihypertensive medication use in patients with hypertension)^38,39^. Our protein findings could help identify vulnerable individuals who are most likely to benefit from interventions aimed at reducing social inequalities by targeting these factors.

While the findings regarding the 14 socially patterned, age-related proteins from the Whitehall study are promising, they require further validation in future research. We successfully replicated associations for 11 of these proteins in an independent cohort study, linking them to various social adversity indicators and mortality; however, data for the remaining 3 proteins were unavailable. Further research is also needed to identify disease profiles for the expanded hallmarks of aging, including impaired macroautophagy, chronic inflammation and dysbiosis, in addition to the original nine hallmarks investigated in our study^33^. In addition, our use of electronic health records shares limitations common to most large multi-outcome cohort studies. These include the inability to detect preclinical and undiagnosed conditions, as well as diseases that rarely result in hospitalization.

Our data originated from countries with distinct social contexts shaped by differences in welfare systems, economic inequality and service access^40,41^. Finland’s welfare state ensures universal healthcare, free education and strong social safety nets, promoting high income equality. The United Kingdom offers a moderately comprehensive system, including the National Health Service (NHS), but faces greater income inequality and entrenched class structures. The United States, with a less comprehensive welfare system, has a dynamic economy but significant disparities between affluent and poorer communities. While data collection methods varied across countries, limiting direct comparisons, the consistent findings across these diverse contexts suggest that our main conclusions may be generalizable beyond the studied populations.

In conclusion, our data support the concept that social disadvantage accelerates the aging process, leading to earlier age-related deterioration in affected individuals. This concept offers a potential explanation for the fundamental role that social stratification plays in determining our health. Our study suggests that these effects may be measurable through plasma and disease profiles.

## Methods

### Study design and participants

We analyzed pseudonymized individual-level data from four prospective cohort studies: the UK Biobank, FPS, Whitehall II and ARIC, with appropriate approvals for access.

The UK Biobank study is a nationwide, prospective cohort study of half of a million participants aged 38–73 years, living in the United Kingdom (https://www.ukbiobank.ac.uk)^42^. Baseline clinical examinations, including measures of social disadvantage, were conducted between 2006 and 2010. Participant follow-up via linked electronic health records was conducted until 2021. Our analyses were conducted using the UK Biobank Resource under application numbers 60565 and 22627. Data collection in the UK Biobank was approved by the North West Multi-Centre Research Ethics Committee. All participants provided written informed consent before their involvement in the study.

The FPS is a prospective occupational cohort study^3^. Study participants included 286,475 men and women aged 40 or older, who were followed up via electronic health records of national health registries until 2016. These registries provide dates and diagnoses of in-patient care in hospitalizations and deaths for all residents in the country. Participants provided electronic informed consent for both baseline assessments and register linkage. The FPS has received ethical approval from the ethical committee of the Helsinki and Uusimaa hospital district (HUS/1210/2016).

The Whitehall study is a prospective occupational cohort study of 10,308 London-based office staff, aged 35–55, with a baseline examination conducted between 1985 and 1988^43^. Blood samples for proteomic analyses were collected for 6,545 participants between 1997 and 1999, all of whom were linked to NHS electronic health records. Written informed consent from participants was obtained at each contact, and ethics approval was obtained from the University College London Hospital Committee on the Ethics of Human Research (reference number 85/0938).

The ARIC study is a population-based cohort study of 15,792 participants aged 45–64 years at baseline (1987–1989), selected through probability sampling from 4 US communities (Washington County, Maryland; Forsyth County, North Carolina; the Minneapolis, Minnesota, suburbs; Jackson, Mississippi)^44^. In proteomic analyses, self-identified non-Black and non-White participants and self-identified Black participants at Washington County and Minneapolis study sites were excluded owing to small sample sizes (*n* = 78 at visit 2 and *n* = 29 at visit 5). Blood samples for proteomic analyses were available for 11,798 participants during visit 2 (1990–1992) and for 5,195 participants during visit 5 (2011–2013). Mortality was ascertained through multiple data sources up until 31 December 2021. The study was approved by each field center’s institutional review board, and all participants provided written informed consent before the study.

A flowchart of sample selection and baseline characteristics of the participants are presented in Extended Data Figs. 1 and 2, and Supplementary Table 1.

### Measurement of social disadvantage

Social disadvantage at baseline was assessed using several indicators: education, father’s occupational status, neighborhood deprivation, occupational position and household income. In the UK Biobank and the Whitehall study, educational attainment was self-reported and classified into three categories: high (college or university degree), intermediate (A levels and AS levels or equivalent, O levels and GCSEs or equivalent, CSEs or equivalent, NVQ or HND or HNC or equivalent, other professional qualifications, for example, nursing, teaching), low (none of the above). In the FPS, information about education was obtained from Statistics Finland via record linkage, including the following three categories: high (tertiary qualification, college or university), intermediate (all other educational qualifications), low (no qualifications, compulsory schooling). In ARIC, self-reported education was categorized as high (any college), intermediate (high school/GED/vocational school) and low (less than high school).

In the UK Biobank and the Whitehall study, neighborhood deprivation was assessed using the Townsend index^45^ linked to the participants’ residential address. This index is calculated from census data on four variables: unemployment (as a percentage of those aged 16 and over who are economically active), non-car ownership (as a percentage of all households), non-home ownership (as a percentage of all households) and household overcrowding. We divided the neighborhood deprivation score into three categories: high (top quartile), intermediate (middle quartiles) and low (bottom quartile).

For each FPS participant, the residential address was linked to a Statistics Finland score for neighborhood deprivation. The score is derived from three components: the proportion of adults with low education, the unemployment rate and the proportion of people living in rented housing within each 250 m by 250 m grid area^3^. As previously, we categorized these data as low (an area deprivation score lower than the national mean), intermediate (from the national mean to the national mean plus 0.5 s.d.) and high (higher than 0.5 s.d.)^3^.

In the ARIC study, neighborhood deprivation was measured using the National Level Area Deprivation Index, which was derived by linking geocoding participant’s geocoded address coordinates with the US Census block group boundaries. Each census block was assigned a rank ranging from 1 to 100, calculated from 18 census-based indicators of socioeconomic disadvantage using the Singh method^46^. We divided the index into tertiles, to represent low, intermediate and high levels of neighborhood deprivation.

In the FPS, information about occupational position was coded based on the International Standard Classification of Occupations (ISCO)^47^ and categorized into three occupational position groups: high (non-manual occupations, ISCO classes 1 and 2, for example, physicians, lawyers), intermediate (non-manual occupations, ISCO classes 3 and 4, for example, registered nurses) and low (service and manual occupations, ISCO classes 5–9, for example, cleaners, maintenance workers). In the Whitehall study, occupational position was obtained from the British civil service occupational grade at baseline, a 3-level variable representing high (administrative), intermediate (professional or executive) and low (clerical or support) grades^43^. In addition, father’s occupational class was assessed with the question ‘What is/was your father’s main job, what kind of work does/did he do in it’. Responses were coded as manual or non-manual.

### PRS for education

The UK Biobank genetic data include genotypes for 488,377 participants, assayed using two very similar genotyping arrays: 807,411 markers using the Applied Biosystems UK BiLEVE Axiom Array by Affymetrix (now part of Thermo Fisher Scientific, for a subset of 49,950 participants) and 825,927 markers using the closely related Applied Biosystems UK Biobank Axiom Array (438,427 participants; shares 95% of marker content with the UK BiLEVE Axiom Array)^48^. In the Whitehall study, genotyping data include the Illumina Human Drug Core Array, which features a whole-genome single-nucleotide polymorphism scaffold with enhanced coverage of 200,000 custom markers in 4,500 drugged or druggable genes^49^.

We constructed a polygenic index for education, as defined in ref. ^21^, to examine whether the associations between social disadvantage, hallmark-related diseases and age-related proteins are attributable to genetic effects across all these constructs.

### Follow-up for ARDs and mortality

UK Biobank and Whitehall participants were linked to the UK NHS Hospital Episode Statistics database for hospital admissions and the NHS Central Registry for mortality. Electronic health records, including dates and the International Classification of Diseases diagnostic codes of hospitalizations and deaths, were retrieved until 2021 in the UK Biobank study and until 2019 in the Whitehall study. The NHS provides most of the healthcare in the country, including in- and out-patient care, and record linkage was undertaken using a unique NHS identifier held by all UK residents. FPS participants were linked by their unique identification number to national hospital discharge (recorded by the Finnish Institute for Health and Welfare) and mortality (recorded by Statistics Finland) registries. These electronic health records included cause (International Classification of Diseases codes) and date of hospitalization or mortality, or both, until 2016.

In the UK Biobank, FPS and Whitehall study, we measured the 4 primary and 5 compensatory and integrative hallmarks of aging indirectly, based on a person’s vulnerability to specific hallmark-related diseases as defined in refs. ^4^^,^^22^. This list comprises a total of 85 diseases, including those 30 diseases most strongly related to each hallmark. These diseases encompass conditions linked to a single hallmark and also those shared by two or more hallmarks. The presence of a specific hallmark of aging is indicated if the participant has one or more diseases related to that hallmark. The 83 diseases and their diagnostic codes for each aging hallmark that were available for this study are listed in Supplementary Table 1 (ref. ^23^).

An extended model of aging hallmarks includes 3 additional hallmarks: disabled macroautophagy (a special case of LOP in the original model), chronic inflammation (a component of AIC in the original model) and dysbiosis (also part of AIC in the original model)^33^. A predefined validated list of diseases related to these 3 new hallmarks was not available at the time of our study.

In the ARIC study, mortality was ascertained through contact with participant proxies via telephone, hospital records, death certificates or vital statistics from the National Death Index until 31 December 2021. As unified national hospitalization registries with comprehensive coverage, such as those in the United Kingdom and Finland, are not available in the United States, similarly high-quality data on hallmark-related diseases were not available in the ARIC study.

### Statistical power and reproducibility

Sample sizes were determined based on available data in the cohorts. As shown in Supplementary Figs. 1 and 2, participants were excluded from the analyses owing to missing data on social disadvantage or hallmark-related diseases. A total of 492,257 participants were included in the analysis of the UK Biobank and 286,475 participants in the analysis of the FPS. In prospective analyses, participants with hallmark-related diseases at or before baseline were additionally excluded. The number of included participants varied between 430,307 and 452,305 in the UK Biobank and between 267,046 and 281,348 in the FPS, depending on the hallmark under investigation (Supplementary Table 5).

Statistical power varied depending on the outcome. For any incident hallmark-related disease in the UK Biobank, we were able to detect a 3–4% difference between high- and low-education groups (hazard ratios 1.03–1.04) at 90% power and an alpha level of *P* = 0.05. The corresponding range for hazard ratios was 6–7% (hazard ratios 1.06–1.07) in the FPS. For specific hallmark-related diseases, the minimally detectable difference in hazard ratios ranged from 1.05 (cataract and osteoarthritis) to 2.65 (essential tremor and scleroderma) in the UK Biobank and from 1.10 (cataract and osteoarthritis) to 3.75 (essential tremor and Barrett’s esophagus) in the FPS.

Reproducibility was assessed using multiple indicators of social disadvantage across four independent cohort studies from the United Kingdom, Finland and the United States. Further examination of reproducibility used methodological triangulation, that is, examining the consistency of the results across alternative epidemiological approaches and sensitivity analyses. The main finding of the social disadvantage–aging hallmark association was consistent across the three independent cohort studies, four alternative indicators of social disadvantage and in multiple alternative analyses.

### Measurement of plasma proteins

Plasma ethylenediaminetetraacetic acid (EDTA) samples drawn at baseline, coinciding with the assessment of social disadvantage, were stored in 0.25-ml aliquots at −80 °C. Proteins were analyzed using the SomaScan version 4.0 and 4.1 assays by SomaLogic, including up to over 7,000 unique proteins. We used the SOMAmer-based capture array, which quantifies the relative concentration of plasma proteins or protein complexes. The SomaScan platform uses short single-stranded DNA with chemically modified nucleotides (modified aptamers) that act as protein-binding reagents with defined three-dimensional structures and unique nucleotide sequences. These aptamers are identifiable and quantifiable using DNA detection technology. This measurement was blinded to participant characteristics, including social disadvantage or hallmark-related disease.

### SomaLogic normalization and quality control

All samples underwent standard SomaLogic normalization, calibration and quality control. To control for batch effects during assay quantification, pooled reference standards and buffer standards are included on each plate. To control for both within-plate and across-plate technical variation, samples are normalized within and across plates using median signal intensities in reference standards. Samples are further normalized to a pooled reference using an adaptive maximum likelihood procedure. If signal intensities deviate significantly from the expected range, samples are additionally flagged by SomaLogic and these samples were excluded from the analysis. SomaLogic provides data on the resulting expression values (the ‘raw’ data).

The scale factor acceptance criterion per plate is between 0.4 and 2.5, and this criterion was passed for all 48 plates for Whitehall EDTA samples. The ‘calibrator percent-in-tails’ refers to the percentage of plate calibration scale factors with values outside the expected range, 0.6–1.4. The alert criterion of 10% was slightly exceeded in 2 of the 48 plates (10.8% and 10.9%, respectively), the values for other plates ranging between 1.7% and 9.6%. The ‘QC percent-in-tails’ refers to the percentage of SOMAmer reagents in the QC control that are outside the accepted accuracy range, 0.8–1.2, when compared with the reference. All plates in the Whitehall study passed the acceptance criteria.

We applied the version 4.0 → version 4.1 multiplication scaling factors provided by SomaLogic to the raw version 4.0 assay expression values to allow direct comparisons across samples analyzed using version 4.0 and version 4.1. The raw data were transformed to a normal distribution by inverse rank-based normal transformation before analysis, as the assay has an expected log-normal distribution.

### SomaLogic probe validation

SomaLogic SomaScan assay technology has been widely used in biomedical research, the list of publications comprising ~700 peer-reviewed papers (https://somalogic.com/publications/). The performance of the SomaScan assay and the modified aptamer binding has been described in detail elsewhere^50,51^. Briefly, there is minimal replicate sample variability (coefficient of variation). The specificity of aptamer reagents is good and has been confirmed in several ways. The median intra- and inter-assay coefficients of variation for SomaScan are ~5%, and assay sensitivity is comparable to that of typical immunoassays, with a median lower limit of detection in the femtomolar range. The majority of SomaScan protein measurements are stable, and a subset of proteins has been validated using laboratory-developed tests. These validated proteins have been delivered from SomaLogic’s Clinical Laboratory Improvement Amendments (CLIA)-certified laboratory to physicians and patients in the context of medical management.

All 7,524 probes on the version 4.1 assay undergo rigorous primary validation of binding and sensitivity to the target protein, including determination of the equilibrium binding affinity dissociation constant, pull-down assay of cognate protein from buffer, demonstration of dose response in the SomaScan assay and estimation of endogenous cognate protein signals in human plasma above the limit of detection. A total of 70% of the SomaScan probes have at least one orthogonal source of validation from mass spectrometry, antibody-based measurements, *cis*-protein quantitative trait loci analysis, absence of binding with the nearest neighbor (that is, no detected signal from the protein that is most closely related in sequence to the cognate protein) or correlation with mRNA levels in cell lines.

### Measurement of organ-specific aging signatures

We computed a total of 9 organ-age gaps, accounting for cohort characteristics, using the ‘organage package’^24^ in Python (https://github.com/hamiltonoh/organage). This package requires SomaScan data version 4.0 or version 4.1, and age and sex as inputs, to compute *z*-scores (mean = 0, s.d. = 1) for organismal and organ-specific age gaps, that is, the biological age of an individual’s organs or body relative to those of same-aged peers. The resulting variables relate to the organ-specific ages of the arteries (14 proteins), brain (202 proteins), heart (10 proteins), immune function (173), intestine (33 proteins), kidneys (12 proteins), liver (113 proteins), lungs (9 proteins) and pancreas (34 proteins), in addition to an overall organismal age (3,907 proteins). The list of proteins included in each age gap is provided in Supplementary Table 9.

### Identification of proteins related to hallmarks of aging

We identified plasma proteins related to each hallmark of aging using the Human Proteomic Atlas (www.proteinatlas.org) with the expanded 65-term taxonomy in ref. ^4^:

- Genomic instability, mtDNA mutations, mtDNA damage, transposable elements, DNA damage, DNA repair deficiencies, mutations, DNA breaks, ssDNA breaks, dsDNA breaks, chromosome breakage;
- Telomere attrition, decreased telomere length, decreased leukocyte telomere length;
- Epigenetic alterations, gene transcription, coding-RNA, noncoding RNA, microRNA, DNA methylation, histone modifications, histone acetylation, histone methylation;
- Loss of proteostasis, endoplasmic reticulum stress, unfolded protein response, proteolysis, proteasome, autophagy, protein aggregation, chaperone;
- Deregulated nutrient sensing, insulin resistance, dyslipidaemia, nutrient sensing pathways, sirtuin 1, Insulin/insulin-like growth factor-1 signaling, mTORC1, AMP-activated protein kinase;
- Mitochondrial dysfunction, mitochondrial toxicity, reactive oxygen species, mitochondrial bioenergetics, electron transport chain, Krebs cycle, mitochondrial dynamics, mitochondrial turnover, mitochondrial degradation, mitochondrial biogenesis;
- Cellular senescence, senescence markers, senescence-associated secretory phenotype (SASP), immune-senescence;
- Stem cell exhaustion, stem cell differentiation, progenitor cell, stem cell self-renewal;
- Altered intercellular communication, inflammatory signaling, inflammaging, inflammation, neural signaling, neurotransmitters, hormonal signaling, hormones.

Details of the search are provided in Supplementary Table 12. To confirm the link with age and controlling for multiple testing, we included only those proteins that were additionally associated with chronological age after Bonferroni adjustment (*P* < 6.58 × 10^−6^), a total of 1,044 proteins (Supplementary Table 13). Of these, 43 were related to SCE, 61 to AIC, 253 to DNS, 11 to CS, 159 to MD, 274 to EA, 1 to TA, 438 to LOP and 296 to GI (Supplementary Table 14).

### Reproducibility of proteomic findings

To confirm the reproducibility of our findings on protein–social disadvantage associations, we repeated the discovery analysis using low occupational position as the indicator of social disadvantage instead of low education; only findings that were replicated across the different indicators of social disadvantage are reported. If multiple aptamers were available to determine a protein hit, we confirmed that the findings were consistent across all the aptamers.

To assess the reproducibility of the associations between proteins and social disadvantage, as well as between proteins and mortality, findings from the UK Whitehall study were validated using an independent dataset from the United States, the ARIC cohort study. Extending the Whitehall findings, the main analyses were repeated separately for individuals with protein measurements taken during midlife and those with measurements taken during old age.

### Statistical approach

In the examination of hallmark-related morbidities, data from the UK Biobank and the FPS were analyzed separately. In a retrospective analysis, we computed the rate of hallmark-specific diseases per 100 person-years for each aging hallmark and examined their rate ratios and 95% CIs by each index of social disadvantage using Poisson regression adjusted for age, sex and ethnicity. We modeled these rates and rate ratios for age 70 in the UK Biobank and for age 55 in the FPS. These were the mean ages at the end of follow-up in these studies.

In prospective analyses of participants with no hallmark-related diseases at baseline, we used Cox proportional hazards models to examine the age-, sex- and ethnicity-adjusted associations of social disadvantage indicators with the first onset of hallmark-related disease within each aging hallmark. To control for genetic confounding, we included PRS for education as an additional covariate in the model.

We used the same approach to examine the age-, sex- and ethnicity-adjusted associations of social disadvantage indicators with the onset of the second ARD among individuals who had already developed one ARD. In addition, we examined the corresponding associations with the onset of the third ARD among those who had already two ARDs.

We calculated clustering coefficients (range 0–1; a higher coefficient indicates stronger connections between the diseases) for groups of diseases that related to a specific hallmark of aging by using the Barrat method of global network transitivity^52^.

We computed phi coefficients to produce a correlation matrix across nine dichotomous aging hallmark variables. For each hallmark, this variable was defined as having at least one versus none of the hallmark-related diseases after baseline.

To examine whether the associations between social disadvantage indicators and the first onset of hallmark-related disease within each hallmark were driven by specific hallmark-related diseases, we analyzed the associations of social disadvantage indicators with all 83 hallmark-related diseases in separate Cox models adjusted for age, sex and ethnicity. To identify the strongest associations, we computed the mean hazard ratio across the two studies and all indicators of social disadvantage weighted by the number of disease cases.

We computed a total of nine organ age gaps, accounting for cohort characteristics, using the ‘organage package’ in Python (https://github.com/hamiltonoh/organage)^24^. This package requires SomaScan data version 4.0 or version 4.1, and age and sex as inputs, to compute *z*-scores (mean = 0, s.d. = 1) for organismal and organ-specific age gaps, that is, the biological age of an individual’s organs or body relative to those of same-age peers.

Before protein discovery analyses were conducted in the Whitehall study, proteins were transformed to a normal distribution using inverse rank-based normal transformation. We included those hallmark-related proteins that were associated with chronological age at proteome-wide significance (0.05/30,000, *P* = 1.67 × 10^−6^) after adjustment for sex and ethnicity. We then examined associations between hallmark-related proteins and indicators of social disadvantage (low occupational status and low education) using logistic regression analysis adjusted for chronological age, sex and ethnicity (White versus non-White) and corrected for multiple testing using the Bonferroni method (*P* = 0.05/1,044, *P* = 4.78 × 10^−5^).

We examined the associations between social disadvantage and the first onset of hallmark-related diseases in the Whitehall study, as was done in the UK Biobank and FPS, including testing for genetic confounding. To investigate the association of change in social disadvantage between early and later adulthood with the levels of proteins, we compared individuals with low education and intermediate or high occupational status (upward social trajectory) with those with low education and low occupational status (persistently low trajectory), as well as individuals with high education and intermediate or low occupational status (downward trajectory) with those with high education and high occupational status (persistently high trajectory). Accumulation of risk was examined using age-, sex- and ethnicity-adjusted Cox models, treating ‘adult life course social standing’ as a five-category exposure and hallmark-related diseases as the outcome.

To confirm the association between hallmark-related proteins and total mortality in both Whitehall and ARIC, we performed a Cox proportional hazards analysis for proteins associated with age and social disadvantage, testing whether these proteins were also linked to mortality after adjustment for age, sex and ethnicity. We report only hallmark-related proteins that were consistently associated with chronological age, mortality and social disadvantage during early (low education) and later life (low occupational status).

To identify protein mediators of the associations between social disadvantage and hallmark-related diseases, we estimated the proportion of this association mediated by social disadvantage-related proteins in a subgroup of people with no hallmark-related diseases at baseline using Cox regression models. Specifically, we used an inverse odds ratio-weighted method to estimate the extent to which the exposure (here social disadvantage) and the mediator (proteins) act as if they are independent of each other, that is, how the exposure directly affects the outcome when excluding the mediator pathway^53^. The inverse odds ratio-weighted method allows simultaneous assessment of multiple mediators, as well as individual mediators, by regressing the set of mediators on the exposure. This approach is particularly well suited for causal mediation analysis in complex models such as ours.

Analyses were performed using R and SAS (9.4), Stata (17.0) and RStudio (2023.03.1). To identify biological processes enriched by the identified 14 proteins, we used the clusterProfiler package and Gene Ontology (GO)-term enrichment analysis in R (ref. ^54^). This approach uses a hypergeometric test and false discovery rate correction for enrichment analyses. To identify a protein interaction network for the 14 proteins, we used the STRINGdb package in R (ref. ^55^). Interactions were searched across the entire String database with a minimum required interaction score of 0.4. Graphs were generated in Excel and the BioRender platform (https://www.biorender.com).

### Reporting summary

Further information on research design is available in the Nature Portfolio Reporting Summary linked to this article.

## Online content

Any methods, additional references, Nature Portfolio reporting summaries, source data, extended data, supplementary information, acknowledgements, peer review information; details of author contributions and competing interests; and statements of data and code availability are available at 10.1038/s41591-025-03563-4.

## Supplementary information

Reporting Summary

Supplementary Data Supplementary Tables 1–34 and statistical code.

## Source data

Source Data Fig. 2 Statistical source data.

Source Data Fig. 3 Statistical source data.

Source Data Fig. 4 Statistical source data.

Source Data Extended Data Fig. 1 Figure and related data.

Source Data Extended Data Fig. 2 Figure and related data.

Source Data Extended Data Fig. 4 Figure and related data.

Source Data Extended Data Fig. 5 Figure and related data.

Source Data Extended Data Fig. 6 Figure and related data.

Source Data Extended Data Fig. 7 Figure and related data.

Source Data Extended Data Fig. 8 Figure and related data.

## Data Availability

Researchers registered with the UK Biobank can apply for access to the individual-level data by completing an application. This must include a summary of the research plan, data fields required, any new data or variables that will be generated and payment to cover the incremental costs of servicing an application (https://www.ukbiobank.ac.uk/enable-your-research/apply-for-access). In the FPS, pseudonymized individual-level questionnaire data can be shared by request to the investigators (jaana.e.pentti@helsinki.fi). Access to linked health records requires separate permission from Findata, the Health and Social Data Permit Authority in Finland (https://findata.fi/en/permits/). Pseudonymized individual-level data from the Whitehall study are available for sharing within the scientific community. Bona fide researchers interested in accessing the pseudonymized data can apply through Dementias Platform UK (https://www.dementiasplatform.uk) or the Whitehall Scientific Committee (https://www.ucl.ac.uk/epidemiology-health-care/research/epidemiology-and-publichealth/research/whitehall-ii/data-sharing). ARIC proteomic data are available through the NHLBI Biologic Specimen and Data Repository Information Coordinating Center (https://biolincc.nhlbi.nih.gov/studies/aric). Additional requests for clinical or proteomic data from individual investigators may be submitted to the ARIC steering committees and will be reviewed to ensure that data can be shared without compromising patient confidentiality or breaching intellectual property restrictions. Participant-level demographic, clinical and proteomic data may be partially restricted based on previously obtained participant consent. Data-sharing restrictions may also be applied to ensure consistency with confidentiality or privacy laws and considerations (https://sites.cscc.unc.edu/aric). Source data are provided with this paper.

## Code availability

All code developed and used throughout this study has been made open source and is available via GitHub at https://github.com/rmjdmj1/SES-ageing.git and in Supplementary Information.
