# Supplementary material for: Social disadvantage accelerates aging
Source: Nat Med. 2025 Mar 14;31(5):1635–43. doi: 10.1038/s41591-025-03563-4 (PMC12092251; doi:10.1038/s41591-025-03563-4)
Supplement: Supplementary file 1 — Reporting Summary [file 41591_2025_3563_MOESM1_ESM.pdf]

nature portfolio

Corresponding author(s): Prof. Mika Kivimäki

Last updated by author(s): Dec 23, 2024

Reporting Summary

Nature Portfolio wishes to improve the reproducibility of the work that we publish. This form provides structure for consistency and transparency in reporting. For further information on Nature Portfolio policies, see our Editorial Policies and the Editorial Policy Checklist.

Statistics

For all statistical analyses, confirm that the following items are present in the figure legend, table legend, main text, or Methods section.

n/a	Confirmed	
☐	☒	The exact sample size (*n*) for each experimental group/condition, given as a discrete number and unit of measurement
☐	☒	A statement on whether measurements were taken from distinct samples or whether the same sample was measured repeatedly
☐	☒	The statistical test(s) used AND whether they are one- or two-sided<br>*Only common tests should be described solely by name; describe more complex techniques in the Methods section.*
☐	☒	A description of all covariates tested
☐	☒	A description of any assumptions or corrections, such as tests of normality and adjustment for multiple comparisons
☐	☒	A full description of the statistical parameters including central tendency (e.g. means) or other basic estimates (e.g. regression coefficient) AND variation (e.g. standard deviation) or associated estimates of uncertainty (e.g. confidence intervals)
☐	☒	For null hypothesis testing, the test statistic (e.g. *F*, *t*, *r*) with confidence intervals, effect sizes, degrees of freedom and *P* value noted<br>*Give P values as exact values whenever suitable.*
☒	☐	For Bayesian analysis, information on the choice of priors and Markov chain Monte Carlo settings
☒	☐	For hierarchical and complex designs, identification of the appropriate level for tests and full reporting of outcomes
☐	☒	Estimates of effect sizes (e.g. Cohen's *d*, Pearson's *r*), indicating how they were calculated

Our web collection on statistics for biologists contains articles on many of the points above.

Software and code

Policy information about availability of computer code

Data collection	No software was used for data collection.
Data analysis	'organage package' in Python (https://github.com/hamiltonoh/organage); RStudio (2023.03.1), including STRINGdb package, clusterProfiler package and GO-term enrichment analysis, SAS statistical software (version 9.4), and Stata (17.0). Statistical code is available in https://github.com/rmjdmj1/SES-ageing.git and Supplementary Information

For manuscripts utilizing custom algorithms or software that are central to the research but not yet described in published literature, software must be made available to editors and reviewers. We strongly encourage code deposition in a community repository (e.g. GitHub). See the Nature Portfolio guidelines for submitting code & software for further information.

Data

Policy information about availability of data

All manuscripts must include a data availability statement. This statement should provide the following information, where applicable:

- Accession codes, unique identifiers, or web links for publicly available datasets
- A description of any restrictions on data availability
- For clinical datasets or third party data, please ensure that the statement adheres to our policy

We used pseudonymised/anonymised individual-level data from four prospective cohort studies: the UK Biobank, the Finnish Public Sector study, the Whitehall II study, and the Atherosclerosis Risk in Communities study. The statistical code is available in https://github.com/rmjdmj1/SES-ageing.git and Supplementary

Information. Extensive summary data are provided in 33 Supplementary tables. Researchers registered with UK Biobank can apply for access to the individual-level data by completing an application. This must include a summary of the research plan, data-fields required, any new data or variables that will be generated, and payment to cover the incremental costs of servicing an application (https://www.ukbiobank.ac.uk/enable-your-research/apply-for-access). In the Finnish Public Sector study, pseudonymised individual-level questionnaire data as used in this study can be shared by request to the investigators (jaana.e.pentti@helsinki.fi). Linked health records require separate permission from the Findata, the Health and Social Data Permit Authority in Finland (https://findata.fi/en/permits/). Pseudonymised individual-level data from the Whitehall study are available for sharing within the scientific community. Bona fide researchers interested in accessing the pseudonymised data can apply through the Dementias Platform UK (https://www.dementiasplatform.uk) or the Whitehall Scientific committee (https://www.ucl.ac.uk/epidemiology-health-care/research/epidemiology-and-publichealth/research/whitehall-ii/data-sharing). ARIC proteomic data are available through the NHLBI Biologic Specimen and Data Repository Information Coordinating Center (https://biolincc.nhlbi.nih.gov/studies/aric). Additional requests for clinical or proteomic data from individual investigators may be submitted to the ARIC steering committees and will be reviewed to ensure that data can be shared without compromising patient confidentiality or breaching intellectual property restrictions. Participant-level demographic, clinical and proteomic data may be partially restricted based on previously obtained participant consent. Data-sharing restrictions may also be applied to ensure consistency with confidentiality or privacy laws and considerations (https://sites.cscc.unc.edu/aric).

Research involving human participants, their data, or biological material

Policy information about studies with human participants or human data. See also policy information about sex, gender (identity/presentation), and sexual orientation and race, ethnicity and racism.

Reporting on sex and gender	Only the term 'sex' is used in this article as we study biological feature. Sex was self-reported. We have reported data disaggregated for sex. Table 1 and Supplementary Tables S1, S5, S22, S26, and S27 provide sex-specific participant numbers and results for main analysis. No significant sex differences were observed.<br>Results on social disadvantage and hallmark-specific ARDs:<br>“These findings were robust, as similar patterns were observed in analyses using an alternative indicator of adult SES—low occupational position—available for FPS participants and in both men and women, although the effects were stronger in men (Supplementary Tables 4, 5).” P. 5, lines 130-132.<br>Results on social disadvantage and plasma proteins:<br>“Sex-stratified analyses showed a similar overall pattern of results in both men and women (Supplementary Table 22).” P. 8, lines 219-220.
Reporting on race, ethnicity, or other socially relevant groupings	We adjust analyses for ethnicity as a potential confounder. Our topic is social disadvantage and for this reason we use education, occupational group and residential social deprivation as our main exposure variables. Results regarding social status are reported in Figures 2–4 and Supplementary Information Tables 2–33.
Population characteristics	In addition to the above, we measured morbidity associated with cellular ageing, the main outcome of this study, plasma proteins, the main mediators in the study, and polygenic risk score for education. Mean age and proportion of men were 57 and 46% in UK Biobank, 46 and 27% in the Finnish Public Sector study, 56 and 71% in the Whitehall study, 57 and 44% in the ARIC middle-aged cohort and 76 and 57% in the ARIC old-age cohort.
Recruitment	The UK Biobank study is a nationwide, prospective cohort study of half of a million participants aged<br>208 between 38 and 73 years, living in the United Kingdom. The Finnish Public Sector study and the Whitehall II study are occupational cohort studies. The prevalence and incidence of disease in occupational cohort studies are lower than in the general population, but the relationships between risk factors and disease are similar. The ARIC study is multi-site, prospective, biracial cohort study. Participants were recruited from 4 U.S. communities: Forsyth County, North Carolina, Jackson, Mississippi, Suburbs of Minneapolis, Minnesota, and Washington County, Maryland.
Ethics oversight	The North-West Multi-Centre Research Ethics Committee, the Ethical Committee of the Helsinki and Uusimaa hospital district, the University College London Hospital Committee on the Ethics of Human Research, and institutional review boards at each ARIC participating center: University of North Carolina at Chapel Hill, Chapel Hill, NC; Wake Forest University, Winston-Salem, NC; Johns Hopkins University, Baltimore, MD; University of Minnesota, Minneapolis, MN; and University of Mississippi Medical Center, Jackson, MS.

Note that full information on the approval of the study protocol must also be provided in the manuscript.

Field-specific reporting

Please select the one below that is the best fit for your research. If you are not sure, read the appropriate sections before making your selection.

☐ Life sciences ☒ Behavioural & social sciences ☐ Ecological, evolutionary & environmental sciences

For a reference copy of the document with all sections, see nature.com/documents/nr-reporting-summary-flat.pdf

Behavioural & social sciences study design

All studies must disclose on these points even when the disclosure is negative.

Study description	Four prospective cohort studies including quantitative data on sociodemographic charateristics, socioeconomic circumstances, plasma proteins, prevalent health conditions at follow-up and incident diseases and mortality at follow-up.
Research sample	We used existing datasets from major well-characterised population-based and occupational cohort studies,containing detailed information on socioeconomic circumstances, health, and plasma proteins. Sample sizes varied between 6544 and 492,257

	depending on the cohort. Mean age and proportion of men were 57 and 46% in UK Biobank, 46 and 27% in the Finnish Public Sector study, 56 and 71% in the Whitehall study, 57 and 44% in the ARIC middle-aged cohort and 76 and 57% in the ARIC old-age cohort. These cohorts are not directly representative of the general adult population.
Sampling strategy	UK Biobank is based on a random sample of the general population. The Finnish Public Sector study and the Whitehall II study included an occupational population from specific workplaces. ARIC is biracial cohort with participants recruited from 4 centres. The cohort studies were selected because of their large sample size and high-quality prospective data on the exposures, biological mediators, and health outcomes of interest. No power calculations were needed given the large sample size. As indicated in our Data Availability section, these datasets are available for bona fide researchers on request.
Data collection	Data collection methods, including questionnaire surveys, clinical examinations, blood analyses, and linkage to electronic health records are described in detail in the manuscript. The authors were blinded to the data collection process.
Timing	Data collection started in 2006 (UK Biobank), 2000 (Finnish Public Sector study), 1997 (Whitehall II), and 1990 (ARIC). Continuous follow-up through linked electronic health records and repeated clinical examinations ended in 2021. There were no gaps in the follow-up.
Data exclusions	Please see Extended data figures 1 and 2. Participants were excluded from the study based on predefined criteria, including missing data on exposure or outcome, non-response, or lack of consent for health record linkage.
Non-participation	Please see Extended data figures 1 and 2. Participation in the cohort studies was voluntary. Among the invited participants (eligible population), non-participation varied between 26% in the Whitehall II study and 95% in the UK Biobank.
Randomization	In this real-life study, participants were not randomized by social disadvantage; differences in covariates were addressed through statistical adjustments. Consistent findings across diverse populations and subgroups further support the robustness of our results.

Reporting for specific materials, systems and methods

We require information from authors about some types of materials, experimental systems and methods used in many studies. Here, indicate whether each material, system or method listed is relevant to your study. If you are not sure if a list item applies to your research, read the appropriate section before selecting a response.

Materials & experimental systems

n/a	Involved in the study
☒	☐ Antibodies
☒	☐ Eukaryotic cell lines
☒	☐ Palaeontology and archaeology
☒	☐ Animals and other organisms
☒	☐ Clinical data
☒	☐ Dual use research of concern
☒	☐ Plants

Methods

n/a	Involved in the study
☒	☐ ChIP-seq
☒	☐ Flow cytometry
☒	☐ MRI-based neuroimaging

Plants

Seed stocks	*Report on the source of all seed stocks or other plant material used. If applicable, state the seed stock centre and catalogue number. If plant specimens were collected from the field, describe the collection location, date and sampling procedures.*
Novel plant genotypes	*Describe the methods by which all novel plant genotypes were produced. This includes those generated by transgenic approaches, gene editing, chemical/radiation-based mutagenesis and hybridization. For transgenic lines, describe the transformation method, the number of independent lines analyzed and the generation upon which experiments were performed. For gene-edited lines, describe the editor used, the endogenous sequence targeted for editing, the targeting guide RNA sequence (if applicable) and how the editor was applied.*
Authentication	*Describe any authentication procedures for each seed stock used or novel genotype generated. Describe any experiments used to assess the effect of a mutation and, where applicable, how potential secondary effects (e.g. second site T-DNA insertions, mosiacism, off-target gene editing) were examined.*
